# Supplementary material for: Vinyl Phosphate-Functionalized, Magnetic, Molecularly-Imprinted Polymeric Microspheres’ Enrichment and Carbon Dots’ Fluorescence-Detection of Organophosphorus Pesticide Residues
Source: Polymers (Basel). 2019 Oct 27;11(11):1770. doi: 10.3390/polym11111770 (PMC6918286; doi:10.3390/polym11111770)
Supplement: Supplementary file 1 [file polymers-11-01770-s001.pdf]

polymers

MDPI

Supplementary Materials

Vinyl phosphate functionalized magnetic molecularly imprinted polymeric microspheres enrichment and carbon dots fluorescence detection of organophosphorus pesticide residues

Supplementary Figures and Tables:

Figure S1. The adsorption kinetics curves of MMIPs and MNIPs at 308 K (a); First-order dynamics simulation of MMIPs (b) and MNIPs (c). Second-order dynamics simulation of MMIPs (d) and MNIPs (e), which depend on Equations (5) and (6), respectively.

Figure S2. Adsorption thermodynamics curves of MMIPs.

Figure S3. Selective recognition curves of MMIPs and MNIPs at 308 K (a); the recycling times of MMIPs' adsorption/desorption to triazophos (b). MMIPs means magnetic molecularly-imprinted polymeric microspheres prepared with triazophos as a template, while MNIPs are prepared without a template.

Figure S4. The magnetization curves of Fe_3O_4 (a), Fe_3O_4@mSiO_2 (b), MMIPs (c), and magnetically controlled separation of MMIPs under the effect of magnets (d).

Figure S5. The effects of molar ratios (a), reaction temperatures (b), and the reaction times (c) of reactants on the stability of CDs synthesized.

Figure S6. The effects of molar ratio (a), reaction temperature (b), and reaction time (c) values of reactants on the stability of CDs@VPA synthesized.

Figure S7. The effects of pH (a) and incubation time (b) on the stability of the CDs@VPA detected.

Figure S8. High resolution XPS spectra of C 1s (a), N 1s (b), and O 1s (c) of CDs.

Table S1. Linear regression data and precision of four organophosphorus compounds in adsorption study.

Table S2. Simulation parameters of pseudo-first-order and pseudo-two-order equations at 308 K.

Table S3. Parameters of Langmuir equation and Freundlich equation.

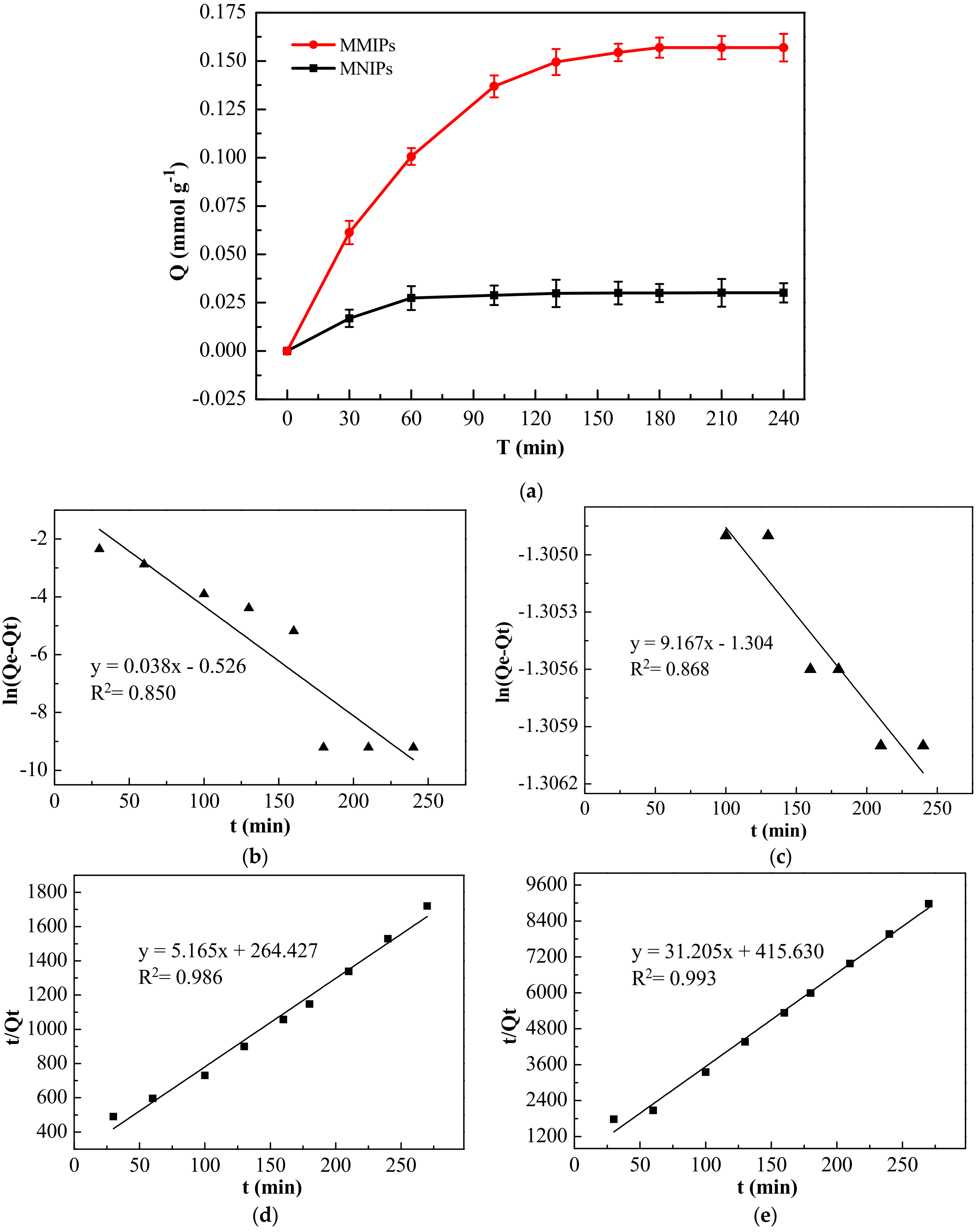

Figure S1. The adsorption kinetics curves of MMIPs and MNIPs at 308 K (a); First-order dynamics simulation of MMIPs (b) and MNIPs (c). Second-order dynamics simulation of MMIPs (d) and MNIPs (e), which depend on Equations (5) and (6), respectively.

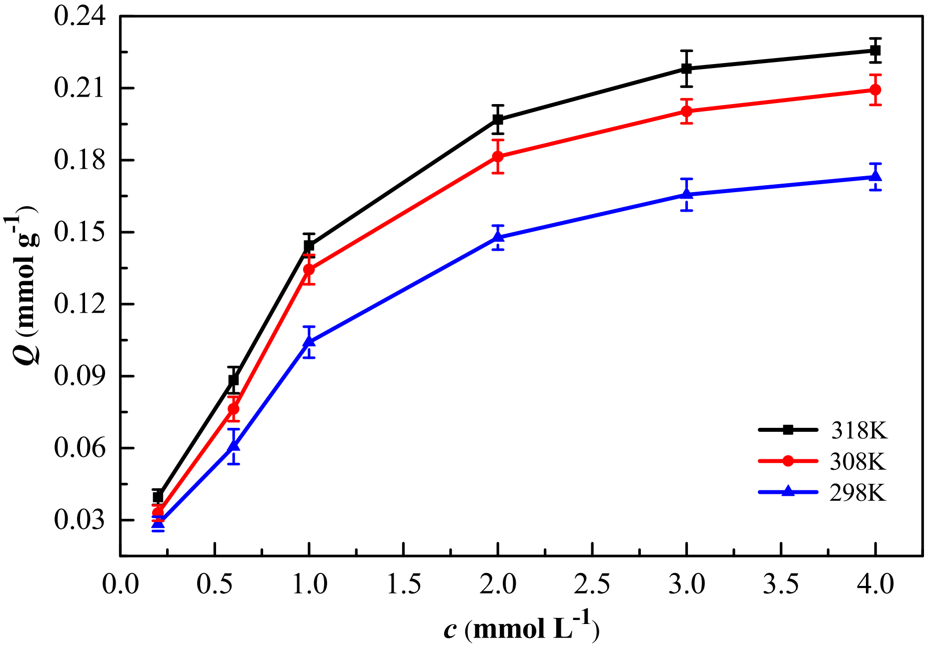

Figure S2. Adsorption thermodynamics curves of MMIPs.

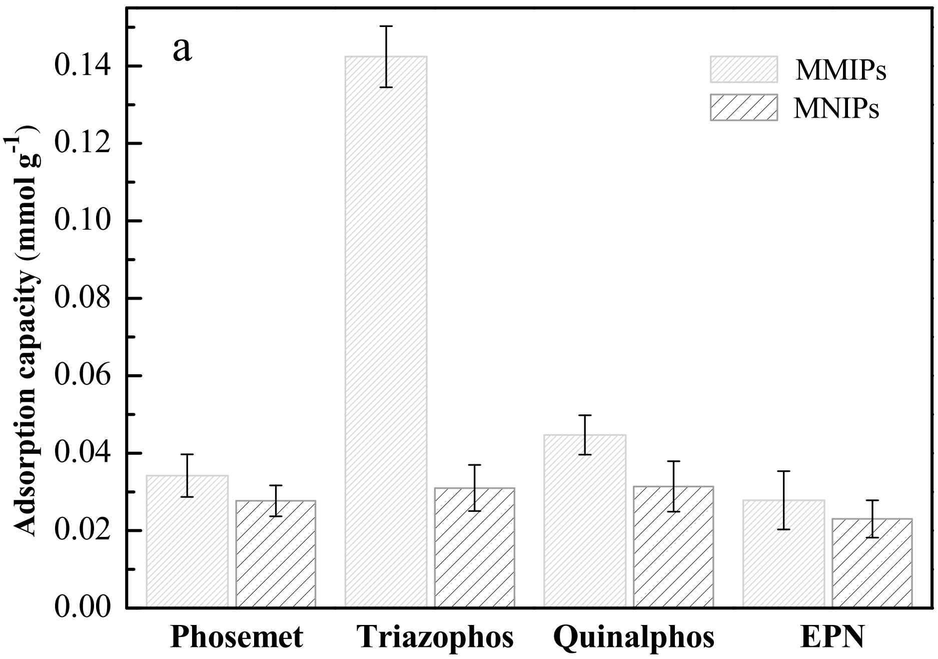

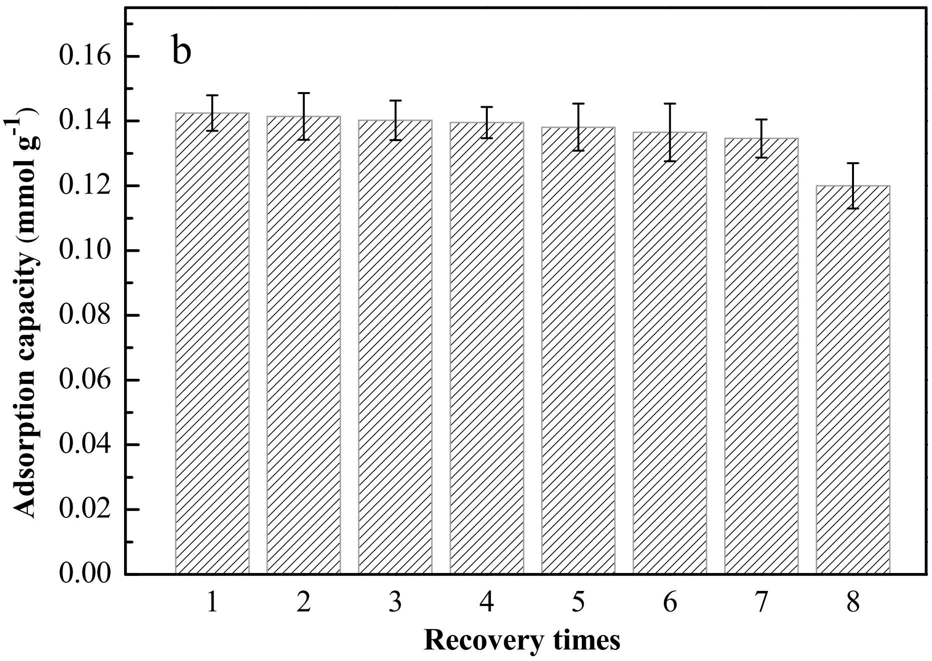

Figure S3. Selective recognition curves of MMIPs and MNIPs at 308 K (a); the recycling times of MMIPs' adsorption/desorption to triazophos (b). MMIPs means magnetic molecularly-imprinted polymeric microspheres prepared with triazophos as a template, while MNIPs are prepared without a template.

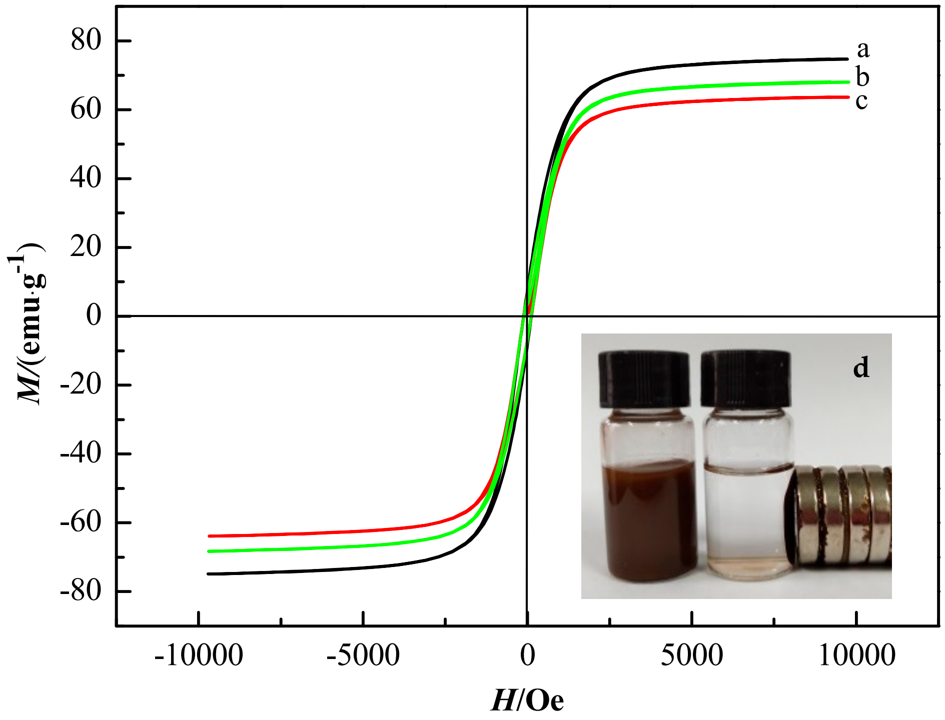

Figure S4. The magnetization curves of Fe_3O_4 (a), Fe_3O_4@mSiO_2 (b), MMIPs (c), and magnetically controlled separation of MMIPs under the effect of magnets (d).

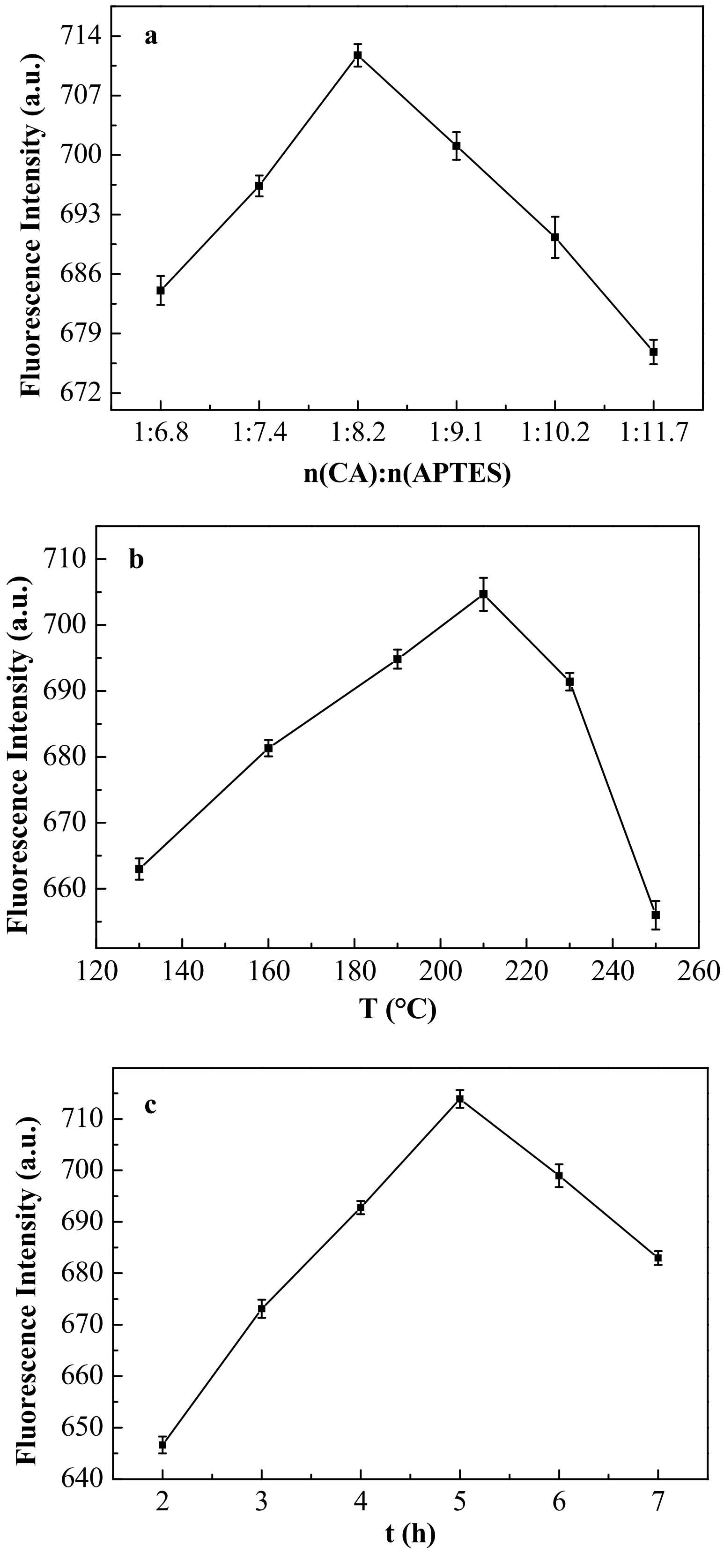

Figure S5. The effects of molar ratios (a), reaction temperatures (b), and the reaction times (c) of reactants on the stability of CDs synthesized.

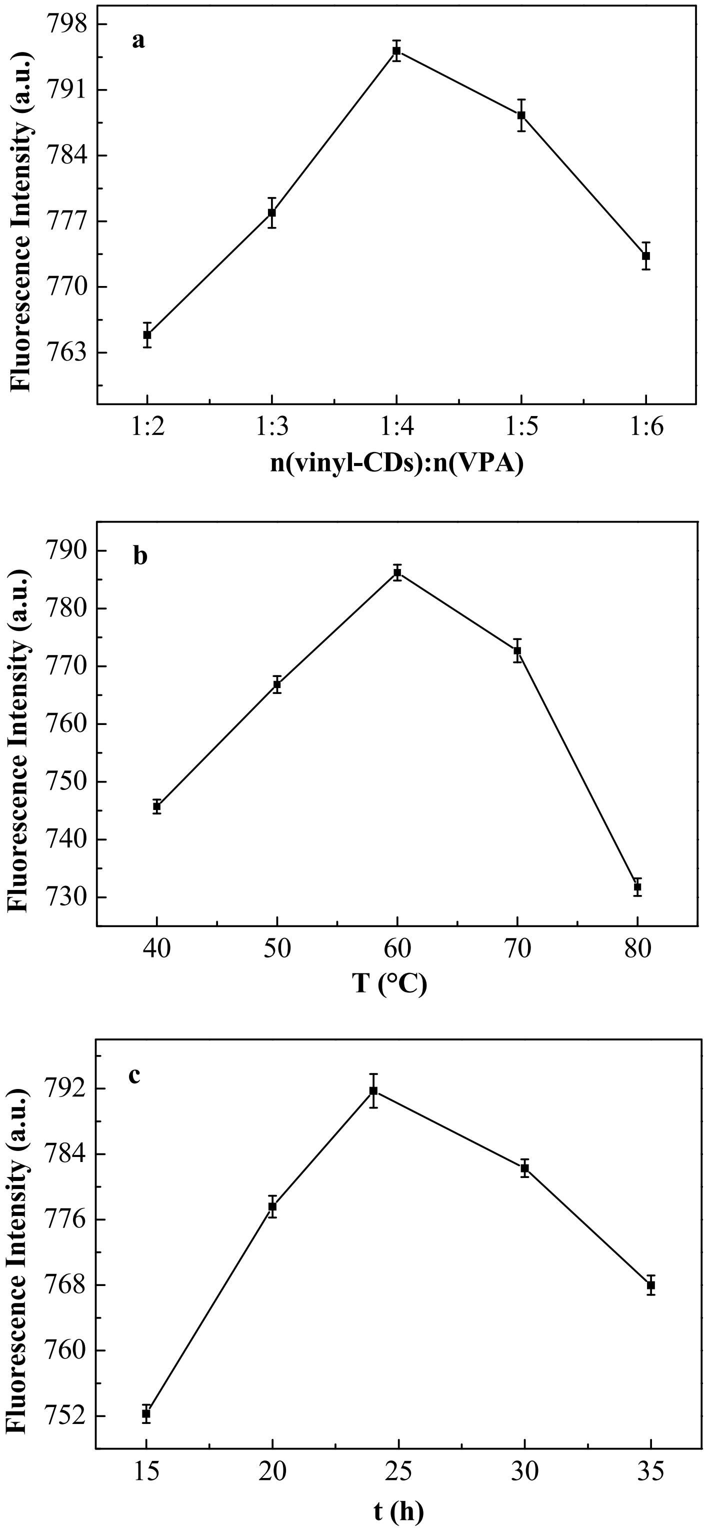

Figure S6. The effects of molar ratio (a), reaction temperature (b), and reaction time (c) values of reactants on the stability of CDs@VPA synthesized.

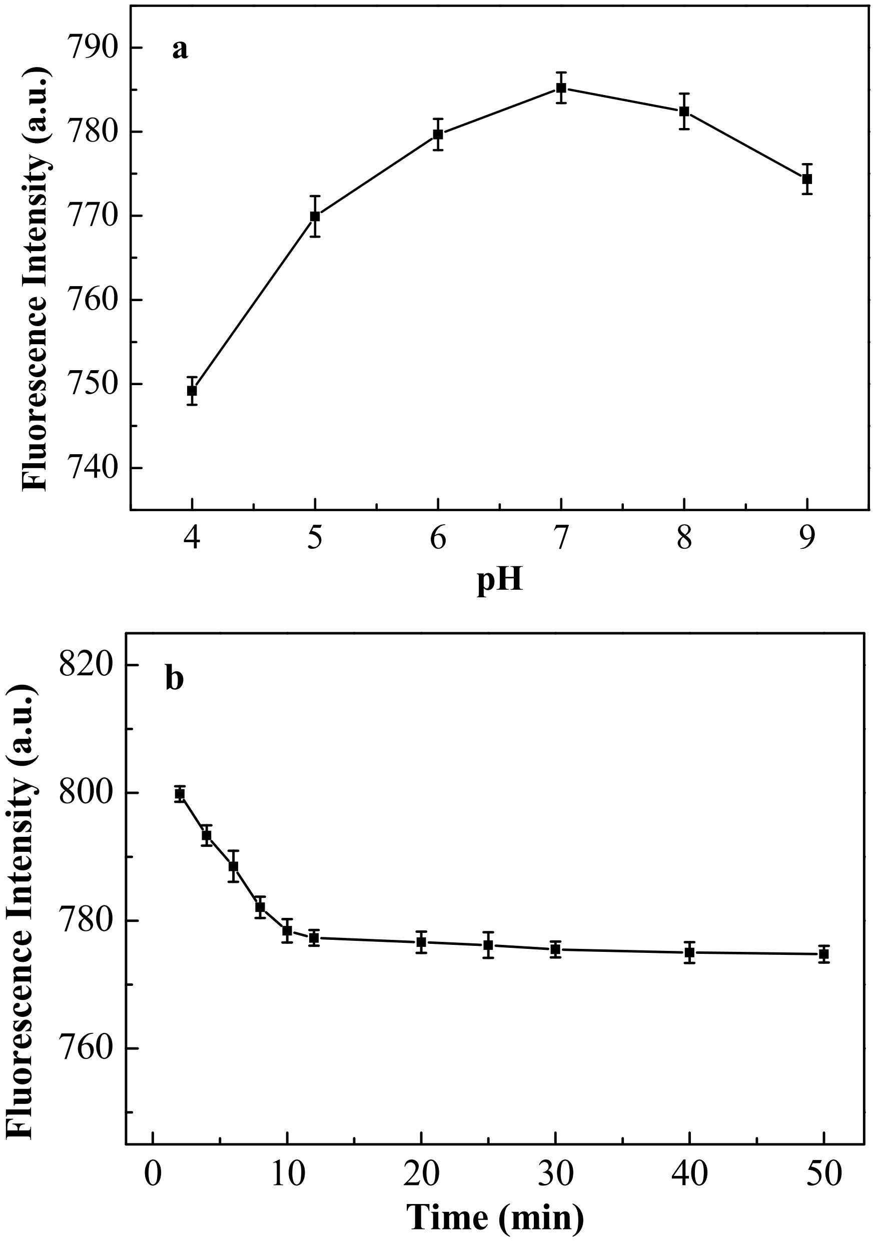

Figure S7. The effects of pH (a) and incubation time (b) on the stability of the CDs@VPA detected.

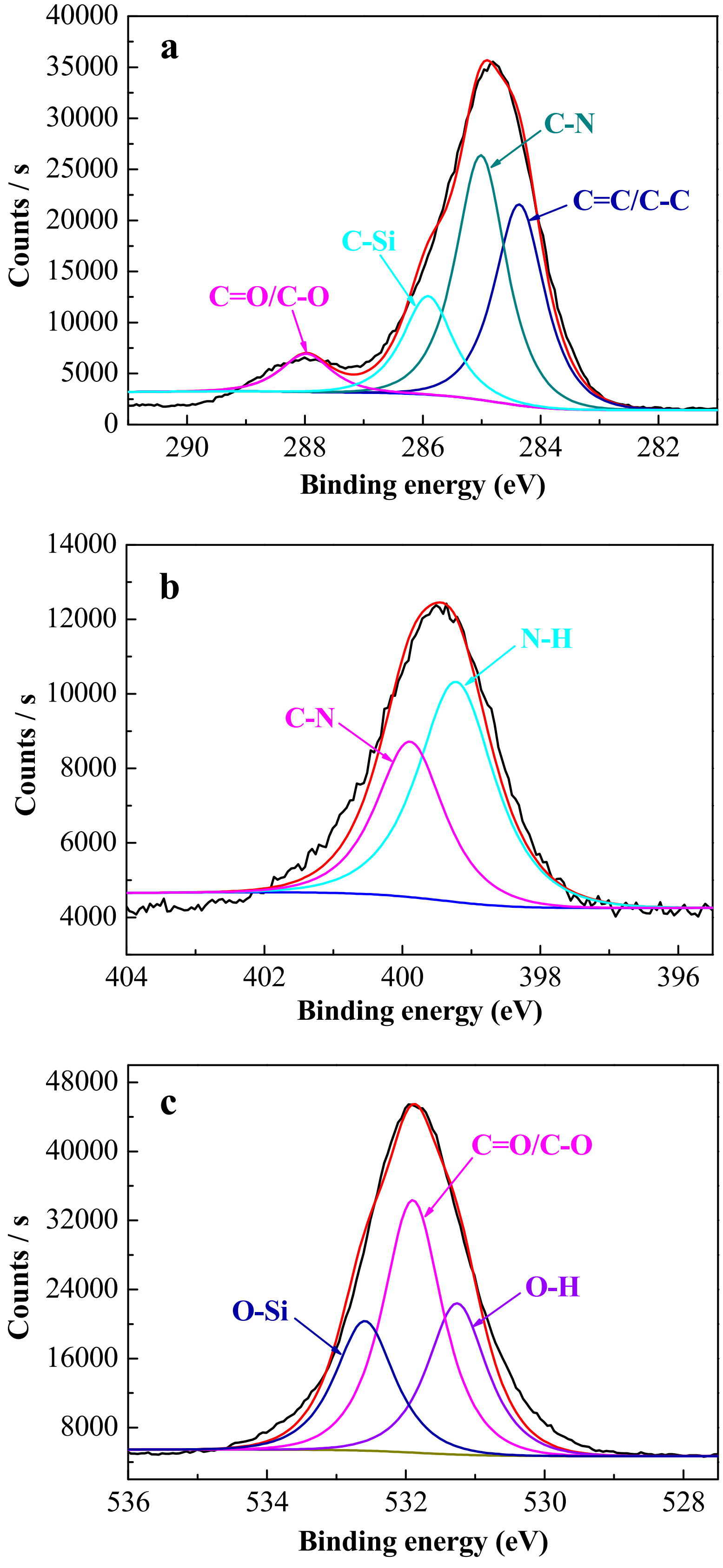

Figure S8. High resolution XPS spectra of C 1s (a), N 1s (b), and O 1s (c) of CDs.

Table S1. Linear regression data and precision of four organophosphorus compounds in adsorption study.

Analytes	Linear regression					Precision
	Regression equations*	R^2	Linear range（mmol L^{-1})	LOD* (mmol L^{-1})	LOQ* (mmol L^{-1})	RSD (%)
Triazophos	$S = 1.15\times10^7$ c + 688485.25	0.9997	6.0×10^{-4}– 5.0	2.0×10^{-6}	1.0×10^{-5}	1.32
Phosemet	$S = 3.78\times10^7$ c + 518222.91	0.9994	1.0×10^{-3}– 3.0	5.0×10^{-6}	1.0×10^{-5}	1.85
Quinalphos	$S = 1.94\times10^7$ c + 289507.68	0.9994	4.0×10^{-4}– 3.5	1.0×10^{-7}	2.0×10^{-6}	2.41
EPN	$S = 9.80\times10^6$ c – 61118.67	0.9992	2.0×10^{-3}– 3.0	6.0×10^{-6}	8.0×10^{-5}	1.39

* Note: S means the peak area (mAu), and *c* is the concentration (mmol L^{-1}) of organophosphorus pesticides. The LOD and LOQ are the concentration of 3 and 10 times the signal-to-noise ratio when the target is detected by HPLC, respectively.

Table S2. Simulation parameters of pseudo-first-order and pseudo-two-order equations at 308 K.

Materials	*Qe*(exptl) (mmol g^{-1})	Pseudo-first-order kinetic model			Pseudo-second-order kinetic model		
		Qe (mmol g^{-1})	k_1 (min^{-1})	R^2	*Qe* (mmol g^{-1})	k_2 (g $mmol^{-1}$ min^{-1})	R^2
MMIPs	0.167±0.014	0.591	0.038	0.850	0.194	0.020	0.986
MNIPs	0.026±0.003	0.271	9.167	0.868	0.032	0.075	0.993

Table S3. Parameters of Langmuir equation and Freundlich equation.

T (K)	Materials	*Qe*(exptl) (mmol g^{-1})	Langmuir isotherm model			Freundlich isotherm model		
			Q_m (mmol g^{-1})	K_L (min^{-1})	R_1^2	K_F (mmol g^{-1})	m (g $mmol^{-1}$ min^{-1})	R_2^2
318	MMIPs	0.226±0.022	0.238	1.250	0.987	0.138	0.600	0.901
308	MMIPs	0.209±0.019	0.220	1.147	0.981	0.126	0.572	0.893
298	MMIPs	0.173+0.015	0.187	1.006	0.981	0.098	0.591	0.913
